# Effects of Place of Articulation Changes on Auditory Neural Activity: A Magnetoencephalography Study

**DOI:** 10.1371/journal.pone.0004452

**Published:** 2009-02-11

**Authors:** Kambiz Tavabi, Ludger Elling, Christian Dobel, Christo Pantev, Pienie Zwitserlood

**Affiliations:** 1 Institute for Biomagnetism and Biosignalanalysis, Malmedyweg, Münster, Germany; 2 Department of Radiology, The Children's Hospital of Philadelphia, Philadelphia, Pennsylvania, United States of America; 3 Department of Psychology, University of Münster, Münster, Germany; Lund University, Sweden

## Abstract

In casual speech, phonemic segments often assimilate such that they adopt features from adjacent segments, a typical feature being their place of articulation within the vocal tract (e.g., labial, coronal, velar). Place assimilation (e.g., from coronal /n/ to labial /m/: rainbow→*raimbow) alters the surface form of words. Listeners' ability to perceptually compensate for such changes seems to depend on the phonemic context, on whether the adjacent segment (e.g., the /b/ in “rainbow”) invites the particular change. Also, some assimilations occur frequently (e.g., /n/→/m/), others are rare (e.g., /m/→/n/). We investigated the effects of place assimilation, its contextual dependency, and its frequency on the strength of auditory evoked mismatch negativity (MMN) responses, using pseudowords. Results from magnetoencephalography (MEG) revealed that the MMN was modulated both by the frequency and contextual appropriateness of assimilations.

## Introduction

Understanding speech involves the mapping of sound onto meaning, a process which presents quite a challenge because of the tremendous variability of the speech signal. This variability is in part due to changes caused by coarticulation of adjacent speech segments. One such process is assimilation, the adoption, by a particular phonemic segment, of features of an adjacent segment. Although it is unclear whether assimilation completely erases the original features, it certainly changes the shape of words. The question is how the speech-processing system deals with such variability.

There is ample evidence that listeners are able to deal with assimilation of adjacent segments [1], [2]. How this is accomplished, and which representations are involved, is still under debate. Therefore, event-related responses revealed by MEG may help elucidate the timing and brain activity underlying compensation for this type of variation in speech. We concentrated on regressive place assimilation, which involves consonants adopting the place of articulation of following consonants. An example is the change from /n/ to /m/, where the coronal phoneme /n/ adopts the labial place of articulation of a following labial (e.g., /green boat/→/greem boat/). For effective comprehension, the resulting change must be compensated for, by perceptual mechanisms [3] and/or by means of flexible representations.

We focus on three issues relevant to compensation for assimilation. The first question, still under debate, concerns the level(s) at which compensation arises. There is evidence for a contribution of early, nonlinguistic auditory processing to compensation for assimilation [3], but which subsequent level is involved: the feature, segmental, lexical level, or multiple levels? Given that segments adopt features from other segments, there is agreement that features must in some way be involved. From there, the proposals diverge. Some argue for a lexical locus of compensation for assimilation [4], [5], while others assume that (abstract) phonemic representations intervene in the process of matching information present in the acoustic-phonetic signal onto abstract word-form representations in the lexicon [6].

The second issue concerns the influence of the phonemic context surrounding assimilated segments. In speaking, assimilation of one segment is elicited by an adjacent context segment whose place of articulation is likely to be adopted. Thus, successful perceptual compensation for place assimilation may well depend on the presence of adjacent segments that elicit and thus license the change (see [1], [3], for overviews). A third issue concerns well-established asymmetries in the frequency with which place features change. While coronal segments often assimilate and adopt a labial or velar place of assimilation, labials and velars almost never surface with a coronal place of articulation [2], [7].

These issues are dealt with differently by the major approaches to compensation for coarticulation (see [1], [2], [5], for overviews). The first assumes a pre-lexical mechanism of *feature parsing* [8], in which feature cues are grouped and assigned to segments. Feature parsing is inherently context-dependent, since it re-aligns parsed features with the adjacent segments they originated from. The mechanism is supposed to be language-independent, and indifferent to the frequency of a particular assimilation. Feature parsing implements compensation at the mapping between features and phonemic segments, not at a lexical level. Note that it runs into trouble with complete assimilation – when all traces of the original feature are lost.

The second position assumes a language-specific mechanism of *phonological inference*, that countermands the effects of assimilation either by rule-application [9], [10], or implemented as compensation learned in a probabilistic connectionist network [11]. This mechanism is context-sensitive, operates at the level of segments (and beyond), and can explain partial and complete assimilation. Because compensation is learned, the mechanism is sensitive to assimilation frequency. It also predicts language-specific effects, for which the evidence is mixed [1], [12].

The third position, the *Featurally Underspecified Lexicon* (FUL), holds that all features are extracted, but not all features are specified in the lexicon [3]. Features are mapped directly onto abstract lexical representations, resulting in either: (i) *match* if both signal and lexicon share the same features (ii) *mismatch* if a feature in the signal contradicts the lexical representation or (iii) *no-mismatch* when an extracted feature is not specified in the lexical representation. Place assimilation of a coronal segment (e.g., /n/, /t/, /d/) results in a no-mismatch, because coronal segments are underspecified for their place of articulation. FUL locates compensation for assimilation at the level of the lexicon, can explain partial and complete assimilation, but is indifferent to context. Given that [coronal] is underspecified but [labial] and [velar] are specified, FUL regards the frequent place assimilation of coronals as legal, and the infrequent velar and labial assimilation as illegal. The model thus predicts clear effects of the frequency asymmetry. Finally, the *tolerance-based account* also locates compensation at the lexical level [4]. While all features are represented with the lexical word-forms, the word-recognition system is more tolerant to minimal than to maximal deviations, and to frequent deviations – for nasal place assimilation, these would be the /n/ to /m/ changes.

To distinguish between these positions, we investigated the consequences and neural correlates of mismatch (and, in terms of FUL, no-mismatch) between features in the signal and phonemic representations, concentrating on nasal consonants. A first question concerns the lowest linguistic level at which compensation for assimilation comes about: At the lexicon, or below? In our understanding, FUL implements compensation for assimilation at the lexical level, assuming underspecified word forms. The same holds for the tolerance-based account. Thus, the input /greem/ can map onto the representation of the word “green”, but what happens when the input is not a word – can /freem/ be “understood” as “freen”? In the feature parsing and inference models, this is possible because compensation is implemented at the level of – adjacent - segments. The use of pseudowords is thus decisive for the level at which compensation comes about.

The second question concerns the phonemic context. In FUL, coronal segments are always underspecified, independent of their position in the word, and of adjacent context (cf. [13]). The contextual appropriateness of the change is thus irrelevant. However, convergent behavioral and electrophysiological evidence suggest that successful compensation relies on the context in which assimilation occurs [cf. 6]–[15]. The third issue concerns the frequency of place assimilation. The only account that predicts no asymmetrical effects is feature parsing (cf. [16]). Table 1 summarizes the predictions made by the main models concerning the three issues addressed here.

**Table 1:** Predictions of the three main approaches with respect to the issues under investigation.

Locus of compensation	FUL	Feature Parsing	Phonological inference
	lexical	pre-lexical	both
Effects of phonetic context	−	+	+
Effects of assimilation frequency	+	−	+

We used MEG to assess phonological variation due to nasal regressive place assimilation, its frequency and contextual appropriateness by means of effects on auditory evoked MMN, whose time course is taken as an electrophysiological index for early speech-comprehension processes [17]. MMN is a neurophysiological index of the detection of a change in the acoustic input that can be elicited in the absence of focused attention [18]). It arises in the oddball paradigm, when listeners are confronted with series of stimuli, some of which are frequently presented (standards) and some infrequently (deviants). Relative to the response evoked by standards, around 200 ms after stimulus onset, deviants evoke a more pronounced response – negative in EEG. This is labelled Mismatch Negativity, MMN.

To address our first question – the locus of perceptual compensation for assimilation – we presented pseudowords in an auditory oddball paradigm. To investigate the frequency asymmetry, we compared two basic conditions. In the first, we presented standards (e.g, *onbo*) with the coronal nasal /n/ and deviants (e.g., *ombo*) with the labial nasal /m/. This deviant constitutes a frequent assimilation of the standard (from /n/ to /m/). If frequency plays a role, we expected this deviant to cause little mismatch. In condition 2, the standard contained the labial nasal (e.g., /m/ in *omdo*), and the deviant contained the coronal nasal (e.g., /n/ in *ondo*). As a consequence of the frequency asymmetry – in line with phonological inference and with underspecification, if it were to apply to a pre-lexical level -, we expected the deviant to cause a clear mismatch to the underlying representation established by the standard. Feature parsing predicts no impact of assimilation frequency.

In conditions 1 and 2, the frequent (n→m) and infrequent (m→n) place assimilations are both followed by a segment that establishes a phonemic context for the change. With two additional conditions, we orthogonally manipulated contextual appropriateness, by contrasting conditions in which the segment following the change from standard to deviant promotes this change (e.g., *onbo*→*ombo*; *omdo*→*ondo*) with conditions in which the context segment is inappropriate for the assimilation (*ondo*→*omdo*; *ombo*→*onbo*). In line with feature-parsing and inference models, and with the bulk of empirical data, we expected an impact of the appropriateness of the phonemic context.

To control for effects due to mere differences between the acoustic properties of standards and deviants, we calculated the “identity” mismatch negativity (iMMN). For this, we subtracted the response to the exact same stimulus presented as deviant and standard across different conditions, thus exploiting the stimulus-status inversion in the oddball paradigm [17], [19], [20].

## Results

**Figure 1:**
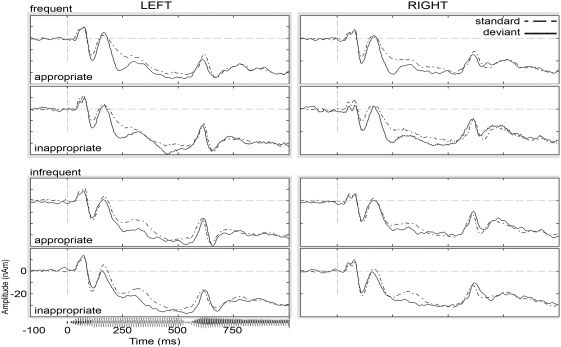
Grand-averaged source waveforms for the experimental odd-ball sequences contrasting frequent/infrequent nasal place feature assimilations embedded in appropriate/inappropriate phonemic context. Robust mismatch activity was present in both hemispheres in all conditions in the interval 170–410 ms following stimulus (shown below the left panel) onset.

The grand average standard and deviant waveforms from each experimental condition are shown in figure 1. With respect to the “traditional” Mismatch, calculated by subtracting standard and deviant from the same condition, the following pattern emerged. In the interval 170–410 ms following stimulus onset, a significant main effect of Assimilation Frequency on mean amplitude (F_(1,15)_ = 8.8, ηp^2^ = 0.4, *p*<0.05) was observed. Infrequent (/m/→/n/) changes resulted in an attenuated MMN response as compared to the frequent (/n/→/m/) changes, the difference between infrequent (−4.0±1.6 nAm) and frequent (−8.3±1.4 nAm) changes amounting to 4.3±1.4 nAm (M±S.E.M). No other effects on MMN activity were observed in this time interval.

Given that the Frequency effect on this MMN activity could be due to physical differences between the standard and deviant stimuli, we computed the identity mismatch, for the same stimulus presented as standard and as deviant. In the post-stimulus interval 170–410 ms, no main effects of Frequency (F<4) and Phonemic Context (F<1) were observed. However, a significant interaction between Frequency and Context (F_(1,15)_ = 13.8, ηp^2^ = 0.5, *p*<0.05) modulated mean iMMN amplitude. Post-hoc examination of the cell means revealed an overall attenuated response to the frequent, contextually appropriate change (−1.6±1.2 nAm), as compared to the frequent-inappropriate (−7.5±2.5 nAm), infrequent-appropriate (−10.2±2.2 nAm), and infrequent-inappropriate (−6.3±1.2 nAm) conditions. The frequent-appropriate condition differed from all other conditions at *p*<0.05 (|t_15_|>2.0), which in turn did not differ amongst each other. It should be noted that the identity of the nasal (/n/ or /m/) can be identified with high levels of confidence some 100–130 ms after stimulus onset –this is based on a separate gating test of the material. Early identification of nasal segments occurs because the initial vowels were not “neutralized” and thus carry information as to the identity of the following nasal. Consequently, information about assimilation frequency, as reflected in the n→m vs. m→n change between standard and deviant, is available quite early (certainly earlier than reflected by the conservative nasal-recognition measure from gating). Thus, when the nasals can be distinguished, the two stimulus types in any given oddball block become uniquely specified, and effects of context (the /d/ or /b/ following the nasal) can come about at the same point in time.

## Discussion

The current study assessed the consequences of variation due to place assimilation on evoked mismatch activity in auditory cortex. With pseudoword stimuli, we investigated the frequency of assimilations along with their licensing by the adjacent segmental context. The data revealed effects of frequency modulated by contextual appropriateness on the mismatch response, as represented here by the iMMN. For the latter, oddball effects are on the exact same speech token, depending on whether it served as standard or as deviant across different stimulation blocks (see Table 1). It was demonstrated earlier that the inversion method of standard/deviant stimuli is sensitive to the feature specification of segments [19], statistical regularities of phoneme clusters [20], as well as lexical items [21].

Our first aim concerned the locus of compensatory mechanisms. Because the results were obtained using pseudowords with no lexical status and no close lexical representations, we argue that our effects reflect auditory processes operating on pre-lexical representations, most probably phonemic segments. This does not fit well with FUL and the tolerance-based account, both of which rely on stored lexical representations to compensate for assimilation. Our data conform to results by Mitterer and colleagues, who observed that the MMN reflects compensation for assimilation even for language material that was foreign to their listeners [12]. Given the pre-lexical locus of the implemented compensatory mechanisms, the feature-parsing and inference approaches can easily accommodate our results. Note that we showed that compensation for assimilation can start early, before lexical access, but this early effect need not be the only compensatory process involved.

Our second aim concerned the impact of contextual appropriateness on the processing of assimilated speech. Underspecification theories such as FUL maintain that the phonemic context which elicits and thus licenses the assimilation is irrelevant, and there is some support for this claim [22]. Context effects that are observed on reaction time are sometimes explained in terms of a frustrated anticipation of appropriate context phonemes [23]. Our present data revealed a clear interaction between context and frequency of change. The differences in iMMN amplitude revealed an asymmetry in neural activity between frequent and infrequent changes – but only if the phonemic context invites the assimilation.

**Figure 2:**
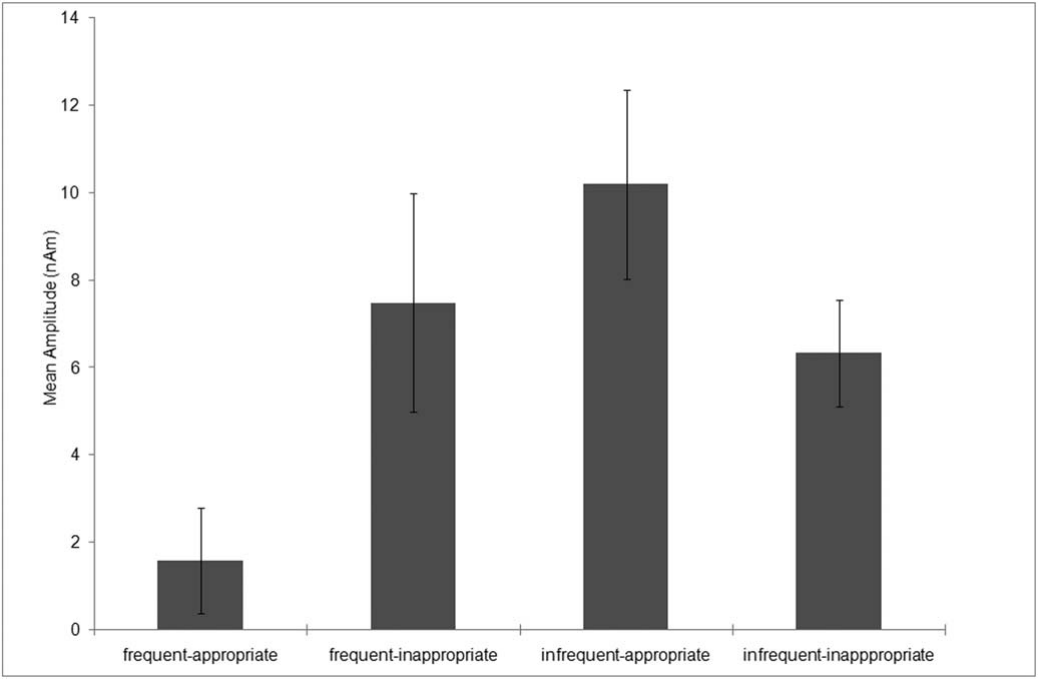
Mean amplitude of the identity mismatch (iMMN) in 170–410 ms post-stimulus interval. The iMMN reflects oddball effects on the same speech token presented as deviant and standard across stimulation blocks. As predicted, an asymmetry in mismatch activity was observed between specified and underspecified segments only for contextually appropriate cases. Compared to the frequent (/n/ to /m/) change from the appropriate context, all other conditions showed significant enhancements in mismatch amplitude. Error bars represent 95% confidence intervals.

Mitterer & Blomert [15] also reported MMN effects as a function of contextual appropriateness of assimilations, using real-word stimuli. In our iMMN data displayed in figure 2, we observed a clear context effect for frequent changes (/n/→/m/), with an attenuated response to contextually appropriate changes relative to inappropriate ones. This corroborates and extends the Mitterer and Blomert findings to show that the lexical level is not relevant for compensation for nasal assimilation. Keep in mind that unlike Mitterer and Blomert, our iMMN analysis is based on a comparison of identical stimuli, in their roles as deviant and standard. As the comparison of our own data from the MMN and iMMN indicate (see results), a direct comparison of our iMMN results to the MMN data by Mitterer and colleagues might be problematic.

With respect to our third question concerning the asymmetry in assimilation frequency (with /n/ to /m/ being frequent and /m/ to /n/ being rare), we indeed observed effects of frequency. This effect was quite pronounced in appropriate contexts, where infrequent changes generated a much larger mismatch response potential than frequent changes. In inappropriate contexts, frequency had no significant impact. Whereas a main effect of assimilation frequency would be in line with the FUL model, the observed interaction between frequency and context is not. Interestingly, iMMN amplitude was enhanced in all cases that implement one or more types of violation, compared to the case that conforms to the subject's knowledge of the patterns governing speech-sound assimilations (the frequent, contextually appropriate condition). Our data are better understood in terms of models that assign a role to both factors. Contextual appropriateness is an inherent feature in the feature parsing and inference models, which can also easily deal with a pre-lexical locus of compensation for assimilation. There is one aspect of our material that is problematic for feature parsing: Given that our speaker pronounced the stimuli as they were written (*ombo*, *onbo*, *omdo*, *ondo*), all changes were complete. This may tip the scale in favour of inference mechanisms, in which early effects of compensation for assimilation are located at the level of adjacent segments.

Although our data provide evidence for early and pre-attentive mechanisms involved in compensation for assimilation, we in no way wish to deny a role for (subsequent) lexical, and even semantic, involvement [2]. Clearly, more research is needed on the timing and neural correlates of all processes underlying the compensatory mechanisms for resolving variability in speech.

In conclusion, the current study provides electrophysiological evidence for early auditory processes involved in speech perception. Auditory evoked mismatch negativity activity was modulated by the frequency and contextual appropriateness of assimilations conveyed by pseudowords. The evidence suggests that early feature extraction from the incoming sensory input provides bottom-up excitation of features, which in turn facilitates phonemic recognition by pattern-matching and automatic change-detection mechanisms in auditory cortex. Assimilation frequency and contextual appropriateness play a role at a phonemic (and thus pre-lexical) level of representation, and beyond, in order to constrain how elements combine into higher-level units such as word forms. Our results provide some backup for underspecification theories, – given that we do observe effects of frequency – and quite some more support for models that envisage a dynamic process of feature extraction, pattern matching and mapping onto segmental representations in a context-dependent way.

## Materials and Methods

Sixteen right-handed German speakers (mean age 24, 11 female) participated in experimental procedures. The subjects gave written informed consent to their participation after they were completely informed about the nature of the study. The Ethics Commission of the University of Münster approved all experimental procedures, which were in accordance with the Declaration of Helsinki.

**Figure 3:**
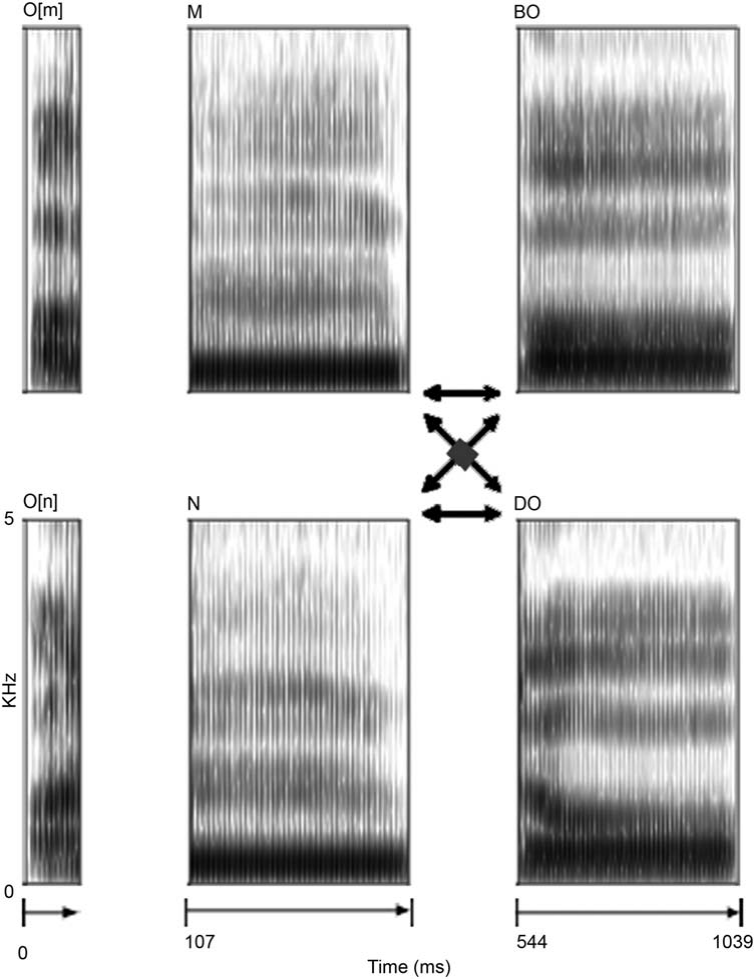
Stimulus material. Disyllabic VC_1_C_2_V pseudowords created by cross-splicing, mixing, and rejoining segments edited from recorded speech.

Disyllabic vowel-consonant-consonant-vowel (VC_1_C_2_V) pseudowords (*ombo*, *omdo*, *onbo*, *ondo*) were quasi-synthesized from digitized recordings by a male native German speaker. Two initial vowels, one from a /m/ context, one from a /n/ context, nasal segments, and second syllables (C_2_V) were edited out and matched for duration. The Pitch Synchronous Overlap Addition algorithm in Praat [24] was used to calculate time windows from the glottal pulses in the original signal. Segment durations were matched by omitting, or appending, overlapping windows using a Gaussian function precisely centred on each glottal pulse. Each nasal segment was neutralized for coarticulatory cues to the identity of the following consonants, by mixing its two realizations in different contexts (/b/ or /d/). Next, the neutralized nasals were recombined with initial corresponding vowels. Finally, the C_2_V (i.e. /bo/ and /do/) were appended at zero crossings, with rising slopes to form 1039 ms long stimuli (see Figure 3). Four different tokens of each pseudoword were created by scaling their pitch contours. The resulting 16 sound files were faded in and out with 50 and 35 ms linear ramps, and the intensity was normalized to mean RMS amplitude across all stimuli.

Table 2 describes the pseudo-random arrangement of stimuli into four odd-ball sequences, with equal numbers of the four tokens per stimulus, separated by a 2.4±0.2 s onset asynchrony, and a 25% probability of deviants occurring. Mismatch always concerned a change in a single place feature of the nasal, embedded in a context that either promoted the assimilation or not (see Table 1). The stimulus-status inversion in the paradigm guaranteed that standard and deviant stimuli were physically identical across conditions. The four different experimental sessions were counterbalanced across participants to counteract order effects.

**Table 2:** Odd-ball paradigm.

	MMNm Condition	Standard	Deviant	Standard	Deviant	iMMNm Condition
1	frequent –appropriate	[onbo]	[ombo]	*(1)* [onbo]	*(4)* [onbo]	infrequent - inappropriate
2	frequent –inappropriate	[ondo]	[omdo]	*(2)* [ondo]	*(3)* [ondo]	infrequent - appropriate
3	infrequent –appropriate	[omdo]	[ondo]	*(3)* [omdo]	*(2)* [omdo]	frequent - inappropriate
4	infrequent –inappropriate	[ombo]	[onbo]	*(4)* [ombo]	*(1)* [ombo]	frequent - appropriate

Odd-ball stimulus pairings used to elicit the auditory mismatch response to assimilation of nasal segments in different phonemic contexts. Mismatch always concerned a change in a single place feature of the nasal, frequent (n→m) and infrequent (m→n), embedded in a context that either promoted the change or not. The identity mismatch (iMMN) was computed by subtracting the evoked response to each pseudoword presented as standard from the response to the same stimulus presented as deviant. The iMMN conditions were defined on the basis of deviants at the time of data acquisition.

Auditory evoked fields were recorded using MEG (275 channel whole-head gradiometer; CTF system Inc., Vancouver, Canada) in a quiet, magnetically shielded room (600 Hz sampling rate, 150-Hz low-pass and 50-Hz notch filters online). For each pseudoword, 1140 ms (100 ms pre- stimulus) epochs were averaged off-line after artefact rejection (threshold 3.0 pT) and off-line noise correction. A DC-offset correction was applied based on the mean value obtained from the pre-stimulus interval.

To estimate the activity in auditory cortex, the method of signal space projection (SSP) [25] was applied to the MEG data, resulting in a virtual sensor maximally sensitive to P50 activity in our subjects. We chose to base the source localization in this study on P50 for the following reasons. First, P50 responses were larger and more reliably detected than the N100 and MMN, thus reducing localization error and affording the best signal-to-noise ratio. Second, it is generally accepted that the MMN is mainly generated in the auditory cortex [26], with overlapping sources for processing complex stimuli [27]. Third, mean P50m and N100m localizations for subjects reliably displaying both components did not differ significantly. For these N = 7 subjects, with bilateral dipole models for both components, localizations did not differ along any of the major axes in 3D space (all |t_6_|<2). Averaged data for each pseudoword stimulus, irrespective of odd-ball status (i.e., standard or deviant) were filtered using 3–150 Hz band-pass for estimating the location of P50m generators. An equivalent single dipole (spatiotemporal model in common stereotaxic space based on individual anatomy) in each hemisphere was approximated to the magnetic field distribution around the maximum (the rising slope) of the global field power between 30 to 80 ms after stimulus onset. Individual models localized within volumes containing Heschl's Gyrus and Planum Temporale [28], [29], and accounting for greater than 90% of residual variance in the measured field were subjected to further analysis. An analysis of variance (ANOVA), with condition as a fixed factor, and with coordinates of acceptable P50m source models for each pseudoword stimulus (irrespective of odd-ball status) as dependent measure, revealed no significant localization differences between conditions (all Fs<2). Individual models meeting the fitting criteria were grand averaged and used as a source model, or SSP “virtual sensor”, used to derive the time course of auditory activity following odd-ball stimuli for each subject.

The resulting source waveforms derived from the SSP “virtual sensor” were band-pass filtered between 0–25 Hz. The “traditional” MMN was obtained by subtracting the response to the standards from that elicited by deviants. The “identity” mismatch negativity (iMMN) was calculated by stimulus-status inversion, by subtracting the response to the same stimulus presented as deviant and standard across different conditions.

## References

[1] Darcy I, Ramus F, Christophe A, Kinzler K, Dupoux E, Kügler F, Fery C, de Vijver R Phonological knowledge in compensation for native and non-native assimilation.. Variation and gradience in phonetics and phonology.

[2] Gaskell G, Snoeren ND (2008). The impact of strong assimilation on the perception of connected speech.. Journal of Experimental Psychology: Human perception and Performance.

[3] Mitterer H, Csépe V, Blomert L (2006). The role of perceptual integration in the recognition of assimilated word forms.. The Quarterly Journal of Experimental Psychology.

[4] Lahiri A, Reetz H, Gussenhoven G, Warner E (2002). Underspecified recognition.. Laboratory Phonology.

[5] Ranbom L, Connine CM (2007). Lexical representation of phonological variation in spoken word recognition.. Journal of Memory and Language.

[6] Frauenfelder UH, Floccia C, Friederici AD (1999). The Recognition of Spoken Words.. Language Comprehension: A Biological Perspective.

[7] Jun J, Hayes B, Kirchner R, Steriade D (2004). Place assimilation.. Phonetically based phonology.

[8] Gow DW (2003). Feature parsing: Feature cue mapping in spoken word recognition.. Perception & Psychophysics.

[9] Marslen-Wilson WD, Nix A, Gaskell G (1995). Phonological variation in lexical access: Abstractness, inference and English place assimilation.. Language and Cognitive Processes.

[10] Gaskell G, Marslen-Wilson WD (1998). Mechanisms of phonological inference in speech perception.. Journal of Experimental Psychology: Human Perception and Performance.

[11] Gaskell G (2003). Modelling regressive and progressive effects of assimilation in speech perception.. Journal of Phonetics.

[12] Mitterer H, Csepe V, Honbolygo F, Blomert L (2006). The recognition of phonologically assimilated words does not depend on specific language experience.. Cognitive Science.

[13] Friedrich C, Eulitz C, Lahiri A (2006). Not every pseudoword disrupts word recognition: an ERP study.. Behavioral and Brain Functions.

[14] Coenen E, Zwitserlood P, Bolte J (2001). Variation and assimilation in German: Consequences for lexical access and representation.. Language and Cognitive Processes.

[15] Mitterer H, Blomert L (2003). Coping with phonological assimilation in speech perception: Evidence for early compensation.. Perception & Psychophysics.

[16] Gow D (2001). Assimilation and anticipation in continuous spoken word recognition.. Journal of Memory and Language.

[17] Pulvermuller F, Shtyrov Y (2006). Language outside the focus of attention: The mismatch negativity as a tool for studying higher cognitive processes.. Progress in Neurobiology.

[18] Näätänen R (2001). The perception of speech sounds by the human brain as reflected by the mismatch negativity (MMN) and its magnetic equivalent (MMNm).. Psychophysiology.

[19] Eulitz C, Lahiri A (2004). Neurobiological Evidence for Abstract Phonological Representations in the Mental Lexicon during Speech Recognition.. J Cogn Neurosci.

[20] Bonte ML, Mitterer H, Zellagui N, Poelmans H, Blomert L (2005). Auditory cortical tuning to statistical regularities in phonology.. Clinical Neurophysiology.

[21] Pulvermuller F, Shtyrov Y, Ilmoniemi RJ, Marslen-Wilson WD (2006). Tracking speech comprehension in space and time.. NeuroImage.

[22] Gumnior H, Zwitserlood P, Bölte J (2005). Assimilation in existing and novel German compounds.. Language and Cognitive Processes.

[23] Lahiri A, Wheeldon L (2000). Phonology: Structure, representation, and process.. Aspects of language production: Studies in cognition series.

[24] Boersma P, Weenink D (2001). PRAAT, a system for doing phonetics by computer.. Glot International.

[25] Tesche CD, Uusitalo MA, Ilmoniemi RJ, Huotilainen M, Kajola M (1995). Signal-space projections of MEG data characterize both distributed and well-localized neuronal sources.. Electroencephalography and Clinical Neurophysiology.

[26] Kujala T, Tervaniemi M, Schroger E (2007). The mismatch negativity in cognitive and clinical neuroscience: Theoretical and methodological considerations.. Biological Psychology.

[27] Takegata R, Paavilainen P, Naatanen R, Winkler I (2001). Preattentive processing of spectral, temporal, and structural characteristics of acoustic regularities: A mismatch negativity study.. Psychophysiology.

[28] Penhune VB, Zatorre RJ, MacDonald JD, Evans AC (1996). Interhemispheric Anatomical Differences in Human Primary Auditory Cortex: Probabilistic Mapping and Volume Measurement from magentic Resonance Scans.. Cereb Cortex.

[29] Westbury CF, Zatorre RJ, Evans AC (1999). Quantifying Variability in the Planum Temporale: A Probability Map.. Cereb Cortex.

